# Translations

**Published:** 1882-05

**Authors:** Seth N. Jordan

**Affiliations:** Columbus, Ga.


### Translations.

By SETH N. JORDAN, M.D., Columbus, Ga.

*From Foreign Journals for the Atlanta Medical Register.*

*Hospital Report. Children's Hospital in Bern. Q. Demme.—Centrablatt, No.* 10, 1882.—In numerous autopsies of diarrhœa patients made during the past summer bacteriæ were found in the blood vessels, in the mesenteric glands, and in the intestinal canal, generally in company with other usual bacteriæ of decomposition. By reason of this Demme has recently introduced for the treatment of summer diarrhœa antiparasitic therapeutics, especially washing out the bowels with a one to two-and-a-half per cent. boracic acid solution, and to combat the collapse, cognac. To relieve tenesmus and the violent peristalsis the repeated introduction of ice suppositories is of great benefit.

The enumeration of the blood corpucles in children nourished with mother's milk was white to red 1:125 (with cow's milk 1:122); the proportion at five months is 1:210, respectively 1:181.

Transfusion of small quantities of blood (5 Grm), often repeated, was practiced in many cases of intestinal catarrh with the best results. Diphtheria is treated with pilocarpine in suitable cases, in conjunction with analeptica, producing good results. Demme holds that the pilocarpine simply aids expectoration, thereby assisting in detaching the false membrane.

Individual cases—Thrombosis of vena cava produced by cheesy degeneration of lymphatic glands pressing on vessel. An antituetic regimen reduced all signs of the swollen veins, with the exception of the caput medusae of the coverings of the bowels.

Bronchial asthma caused by urolithiasis, in child 9 years of age, which disappeared after 11 small stones composed of urates had been passed. Only milk diet was allowed for 9 days before the passage of the stones, and during which time the urine was loaded with urates.

In a case of fat diarrhœa with fatal termination in a child eleven years old, a congenital atrophy of the pancreas was found.

Malignant lympho-sarcoma of the axilla in a girl eleven years old, with lucæmia and numerous metastatic tumors.

Two cases of empyema were successfully treated by resection of the ribs. In a child 5 years old who had swallowed a brass button, a perityphlitis developed and burst into the rectum. Passage of foreign body followed by recovery.

Strumitis in a newborn babe, which died on the day after birth.

---

*The Connection Between Diabetes and Diseases of the Uterus. M. Loeb, Med. Wochenschrift, Berlin,* 1881, *No.* 41. *Diabetic Neuralgia. Drasche, Med. Wochenschr.*, 1882, *No.* 1 and 2.—(1.) The occurrence of diabetes in connection with diseases of the female genitals has been conspicuous to M. Loeb, and he conceives their relation thus: that from the genitals, especially the uterus, the spinal cord in a reflex way is brought into sympathy, as in the case of reflex paralysis, and thus causing diabetes, since numerous experiments and clinical experiences point to the cord as the starting point of diabetes.

(2.) Drasche communicates two cases of neuralgia in diabetic patients, analogous to many other similar cases of recent observation. The first case, a man 64 years old, troubled with intercostal neuralgia, which became especially violent after each meal, and had opposed all manner of treatment for ten years, was perfectly cured as soon as the diabetes became known, a corresponding regimen having been ordered. In another male patient, about 60 years of age, a similar favorable result was arrived at. This patient, in neglecting diet, was annoyed with neuralgia in the regions of both brachial plexus, and the left N. ischiadicus, and on the appearance of the neuralgia the amount of sugar excreted increased. Proper diet reduced both.

---

*On the Treatment of Hyperæmia of the Brain. M. Buch, Centralblatt, No.* 11, 1882.—Supported by the experiments of various investigators, concerning the influence of irritation of the skin on the calibre of the blood-vessels of the brain, and pia mater, etc., B. recommends irritation of the skin in the treatment of hyperæmic conditions of the brain and its coverings. In those cases reported at much length by the author, the patients complained of headache, fullness, stars before the eyes, etc.

The manner in which skin irritation is produced is by pricking small spots with a needle and rubbing in daily equal parts of ol. therebinth. gallicum, ol. tiglii, and is known as Baunscheidt's method.

---

*Syphilis and Irritation. Finger. Med. Wochenschrift. Berlin. No.* 41, 1881.—As further proof that syphilis attacks such places on the skin as are already irritated, let the following serve: Constitutional syphilide developed itself in a patient of the Vienna clinique, who was suffering with herpes tonsurans of the knee, in form of a specific efflorencence, and first in those places still irritated by a slight eczema; during which time the skin of the rest of the body remained free. In another instance a lenticular-papulous return appeared only on such parts as had recently been irritated by artificial eczema produced by iodoform collodion.

---

*Rare Tumors. Weichselbaum, Virchow's Archives, LXXXV, p.* 554.—1. Ganglionic neuroma of kidney, size of a cherry, which consisted for the most part of nerve fibers, with deposits of ganglionic cells.

2. Papillary adeno-sarcoma of the lung, round and large as a hazelnut, lying close to the pleura, and consisted chiefly of vascular papillæ.

3. Primary fibro-sarcoma on the surface of the spleen, and primary multiple endothelium—sarcoma of the spleen.

4. Lymphoma of the spleen.

---

*Skin Eruptions following Vaccination. G. Behrend, Med. Wochensch., Berlin*, 1881, *No.* 46.—Such eruptions as above are not specifically due to the vaccine. Against such a view argue the manifold shape, variation in duration, etc. Again, similar eruptions are often observed after the administration of other medicaments, and following septic infection.

---

*Psoriasis. E. Sesemann, Wochenschrift.* 1881. *No.* 44.—S. uses a mixture of 10 grm. chrysarobine and 4 grm. of collodion. The affected part is treated every other day, a bath given every two weeks. When the skin is very dry, anoint with lard. New spots must be touched up daily with the mixture.

---

*Dyspepsia in Nephritis. J. Thomayer, Centralblatt*, 1882, *No.* 10.—Thomayer has found that in many cases of nephritis the attendant dyspepsia is solely due to a genuine interstitial gastritis; but it is impossible to derive this inflammation direct from the kidneys, or from the uræmia.

